# Positive correlation between circulating cathelicidin antimicrobial peptide (hCAP18/LL-37) and 25-hydroxyvitamin D levels in healthy adults

**DOI:** 10.1186/1756-0500-5-575

**Published:** 2012-10-24

**Authors:** Brian M Dixon, Tyler Barker, Toni McKinnon, John Cuomo, Balz Frei, Niels Borregaard, Adrian F Gombart

**Affiliations:** 1 USANA Health Sciences, Inc, 3838 West Parkway Boulevard, Salt Lake City, UT 84120, USA; 2 Sport Science Department, The Orthopedic Specialty Hospital, Murray, UT 84107, USA; 3 Linus Pauling Institute and Department of Biochemistry and Biophysics, Oregon State University, Corvallis, OR 97331, USA; 4 The Granulocyte Research Laboratory, Department of Hematology, National University Hospital, Copenhagen, Denmark

**Keywords:** Vitamin D, 25-hydroxyvitamin D, Cathelicidin, hCAP18, LL-37, Immunity, Serum, Plasma, CAMP, Infection

## Abstract

**Background:**

Transcription of the cathelicidin antimicrobial peptide (*CAMP*) gene is induced by binding of the bioactive form of vitamin D, 1,25-dihydroxyvitamin D, to the vitamin D receptor. Significant levels of the protein hCAP18/LL-37 are found in the blood and may protect against infection and/or sepsis. We hypothesized that serum vitamin D levels may modulate the circulating levels of hCAP18. Only three studies have shown a positive correlation between circulating 25-hydroxyvitamin D and hCAP18 levels. Here we provide additional evidence for such a correlation in healthy, middle-aged adults.

**Findings:**

Serum levels of 25-hydroxyvitamin D [25(OH)D] and plasma levels of hCAP18 were determined in 19 healthy middle-aged (mean of 50.1 years) adult men and women. Plasma hCAP18 concentrations correlated with serum 25(OH)D concentrations in subjects with 25(OH)D levels ≤ 32 ng/ml (r = 0.81, p < 0.005) but not in subjects with concentrations > 32 ng/ml (r = 0.19, p = 0.63).

**Conclusions:**

We conclude that plasma hCAP18 levels correlate with serum 25(OH)D levels in subjects with concentrations of 25(OH)D ≤ 32 ng/ml as opposed to those with concentrations > 32 ng/ml and that vitamin D status may regulate systemic levels of hCAP18/LL-37.

## Findings

### Background

*CAMP* gene expression is important in host defense as mice and humans lacking hCAP18/LL-37 are susceptible to bacterial infections in numerous tissues including the skin, eye, urinary tract, colon and lung [1-11]. Also, increasing hCAP18/LL-37 in these tissues helps reduce and clear infection [12-14]. Collectively, these data strongly implicate expression of the *CAMP* gene in maintaining adequate host defense. In addition, the LL-37 peptide has additional biological activities that include neutralizing LPS, chemoattraction of leukocytes and promotion of wound healing and angiogenesis [15].

The human cathelicidin antimicrobial peptide (*CAMP*) gene encodes an 18 kDa propeptide (hCAP18) that is packaged in secondary granules of neutrophils and proteolytically cleaved to generate the mature antimicrobial peptide, LL-37, that kills invading pathogens [16-19]. The *CAMP* gene is regulated at the level of transcription by the bioactive form of vitamin D, 1,25(OH)_2_D, and the vitamin D receptor [20-22]. The hCAP18/LL-37 protein is expressed by a wide array of immune cells [23,24] and tissues that are constantly exposed to microbes including the skin, mouth, airways, intestine and colon and is secreted in saliva, sweat, semen and circulates at high levels in the plasma [25-30]. *In vitro* administration of 1,25(OH)_2_D induces *CAMP* gene expression in immune and other epithelial barrier cells [20-22,31,32]. It has been shown that adequate levels of serum 25(OH)D are required for expression of hCAP18/LL-37 by immune-activated macrophages [33], but the relationship between serum 25(OH)D and plasma hCAP18/LL-37 levels has been unclear [34,35].

The biological importance of circulating hCAP18/LL-37 is not well understood, but it may play a role in protecting against infection and/or the development of sepsis [20]. Recently we found that dialysis patients with the lowest levels of hCAP18 were at a greater than 2-fold risk of death of infectious causes, but a correlation between 25(OH)D levels and hCAP18 was not observed [34]. Also, in a study of patients with bone disease we did not find a correlation [34,35]. On the other hand, severely ill (sepsis and non-sepsis) patients were found to have lower 25(OH)D and LL-37 than healthy subjects and a positive correlation between vitamin D levels and LL-37 was shown in all populations [36]. More recently, Bhan and colleagues demonstrated that 25(OH)D levels positively correlate with both baseline cathelicidin levels and changes in cathelicidin levels after high-dose ergocalciferol treatment [37]. Interestingly, the positive correlation only existed at 25(OH)D levels <32 ng/ml, but not when the levels were >32 ng/ml [37]. The relationship between vitamin D status and circulating hCAP18 levels has been inconsistent; therefore, we conducted a prospective study in healthy middle-aged adults to test the hypothesis that serum vitamin D modulates circulating hCAP18 levels.

### Materials and methods

#### Subjects

A total of 19 healthy, volunteer adults were recruited from the greater Salt Lake City metropolitan area (~40° North latitude). The gender and racial composition, age, vitamin D intake from a multivitamin and/or dietary sources and average serum 25(OH)D levels are summarized in Table 1. All subjects were known to be free of disease and were not taking medication or a stand-alone vitamin D product, but could have been taking a multivitamin with vitamin D or other supplements that do not contain vitamin D. All serum and plasma were collected from each individual during the month of December. Written informed consent for participation in the study was obtained from participants. The protocol was approved by the ethics review committees at USANA and OSU.

**Table 1:** Characteristics of subjects enrolled (n = 19)

**Subjects**	**Male/Female**	**Mean age**	Ethnicity**	**Mean Vitamin D intake (IU)**	**Mean serum 25(OH)D (ng/ml)**
**19**	10/9	50.1	16C/2A/1P	602.6 ± 362.3	33.4 ± 13.9

*C – Caucasian; A – Asian; P – Pacific Islander.

± Standard deviations indicated.

#### 25(OH)D assay

To determine circulating 25-hydroxyvitamin D levels a modified technique for vitamin D extraction and measurement was developed [38,39]. Briefly, 100 μl of serum was added to 400 μl of a 2:1 methanol:chloroform solution containing deuterated 25-hydroxyvitamin D3 as an internal standard (40 ng/ml final concentration). To achieve phase separation, 750 μl of ddH2O was added, mixed by vortexer, and centrifuged at 15,000 g for 2 min. The aqueous phase and debris between the two phases were removed, discarded, and the remaining solution dried to completion. The pellet was dissolved in 100 μl methanol and analyzed by injecting 10 μl into an Agilent HPLC (Series 6410, Model G6410A, Santa Clara, CA, USA). 25(OH)D_3_, 25(OH)D_2_ and the internal standard were detected on an Agilent tandem mass spectrometer (Series 6410, Model G6410A, Santa Clara, CA, USA) using atmospheric pressure chemical ionization (APCI) detection. Concentrations of 25(OH)D_3_, 25(OH)D_2_ and the internal standard were determined relative to authentic standards and corrected for recovery. Average recovery was 92.2%. Intra-day coefficient of variation was 4.7% and inter-day coefficient of variation was 9.4%. The detection limit was determined to be less than 1 ng/ml of all analytes. The results were directly compared with two external, FDA-approved, clinical laboratories (Quest Diagnostics and ZRT Laboratory, Beaverton, OR) and levels determined by our assay matched their results (data not shown).

#### Determination of circulating protein levels

The levels of hCAP18 (ng/ml) in each plasma sample were determined using a non-commercial ELISA with a detection limit of 0.084 ng/ml and an intra- and inter-assay coefficient of variation of ≤6.3% [30].

#### Statistical analysis

Data was checked for normality with a Kolmogorov-Smirnov test prior to all statistical analyses. Relationships between variables were examined with a Pearson Product Moment Linear correlation. All statistical analyses were performed with SysStat software (SigmaPlot 10.0, SigmaStat 3.5, Chicago, IL). Statistical Significance was set at p < 0.05 (for correlation coefficients, n = 10, r ≥ 0.632; n = 9, r ≥ 0.666).

### Results

**Figure 1:**
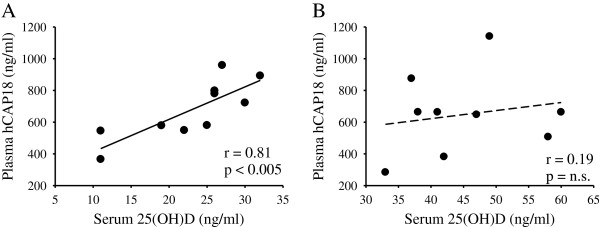
**Correlation between serum 25(OH)D and plasma hCAP18 levels.** Plasma hCAP18 concentrations (ng/ml) correlated with serum 25(OH)D concentrations (ng/ml) in subjects with 25(OH)D ≤ 32 ng/ml (panel **A**, n = 10, solid regression line) but not in subjects with concentrations > 32 ng/ml (panel **B**, n = 9, dotted regression line).

When analyzed as one group (n = 19; mean age 50.1 ± 8.4), no statistically significant correlation between serum levels of 25(OH)D and plasma hCAP18 was identified (data not shown). Bhan and colleagues reported a positive correlation only existed at 25(OH)D levels <32 ng/ml, but not with higher levels [37]. Therefore, subjects were separated into two groups based on serum 25(OH)D levels: those with levels ≤ 32 ng/ml and those above. Interestingly, plasma hCAP18 concentrations strongly correlated with serum 25(OH)D concentrations in subjects with 25(OH)D levels ≤ 32 ng/ml (r = 0.81, p < 0.005) as compared to subjects with concentrations > 32 ng/ml (r = 0.19, p = 0.63) (Figure 1 A & B). When the cut-off was set at serum levels of 25(OH)D less than 40 ng/ml, the correlation between 25(OH)D and hCAP18 in subjects was non-significant ( r = 0.43, p = 0.14). It was not possible to test cut-offs at serum levels of 25(OH)D less than 25 or 20 ng/ml as the number of subjects were too low for statistical power. 

### Discussion

With the discovery that bio-active forms of vitamin D induce the expression of the *CAMP* gene, it has been hypothesized that vitamin D status may affect the levels of circulating hCAP18 [20,34]. In a study of dialysis patients, those with the lowest circulating levels of hCAP18 were at a greater than two-fold increased risk of death of infectious causes, but a correlation between serum 25(OH)D and hCAP18 levels was not observed [34].

More recently, Bhan and colleagues discovered a positive correlation in healthy individuals (mean age 39) at 25(OH)D levels <32 ng/ml, but not when levels were higher [37]. In their study, the 25(OH)D and cathelicidin concentrations were rank transformed, leaving a possible question as to the biological validity and interpretability of the association. On the other hand, Jeng and colleagues observed a positive association between vitamin D status and plasma LL-37 in sepsis, critically ill and healthy patients without transforming the data [36]. Interestingly, 100% of the sepsis patients (mean age 54.0 ±17.1), 92% of the critically ill patients (mean age 56.1 ±15.9) and 66.5% of the healthy individuals (mean age 46.5 ±6.1) had serum 25(OH)D levels below 30 ng/ml [36]. The authors did not apply a cut-off for analysis, but the majority of their subjects were already below 32 ng/ml, and it is possible that analysis of subjects >32 ng/ml 25(OH)D would have lacked the statistical power to discern such a trend. Similarly, in a study by Alvarez-Rodriguez and colleagues of 71 healthy individuals with about two-thirds of participants below 32 ng/ml serum 25(OH)D, a positive correlation between serum 25(OH)D and LL-37 levels was observed [40]. On the other hand, no relationship between maternal 25(OH)D serum levels and LL-37 were detected in cord-blood samples [41] or in patients with active pulmonary tuberculosis [42], but again these studies did not apply a cut-off in the analysis. Interestingly, Alvarez-Rodriguez and colleagues showed that LL-37 levels decreased with age [40]; however, it also has been reported that LL-37 levels increase with age [43,44]. A correlation with age could not be examined in this study due to the limited numbers of subjects.

This study confirms the findings of the three earlier studies described above [36,37,40] and further confirms those of Bhan and colleagues [37] that plasma hCAP18 levels correlated with serum 25(OH)D in subjects with 25(OH)D concentrations ≤ 32 ng/ml as opposed to those with concentrations > 32 ng/ml. These studies suggest that there is a threshold for the biological effects of vitamin D, as measured by 25(OH)D, at or around 32 ng/ml particularly with regard to hCAP18/LL-37 blood levels. To determine the exact threshold (and optimal circulating level of 25(OH)D) for each downstream effect of vitamin D, further research needs to be conducted. Studies with much larger subject numbers and with a broad range of serum 25(OH)D levels would allow testing of different thresholds to determine if the cutoff is higher or lower than 30–32 ng/ml. This study did not show a correlation at a cutoff of 40 ng/ml 25(OH)D, but we could not test lower cutoffs due to insufficient subject numbers.

Given the nature of the relationship between serum 25(OH)D and hCAP18/LL-37 levels, it is possible that vitamin D supplementation or exposure to sunlight to synthesize vitamin D may provide a means to raise systemic levels of hCAP18/LL-37, thus enhancing protection against infection and/or sepsis. In support of this, *in vivo* supplementation of individuals with serum 25(OH)D levels <32 ng/ml resulted in an increase in hCAP18 levels in those individuals showing the greatest increase in serum 25(OH)D [37]. In another study, supplementation of normal and atopic dermatitis (AD) patients with 4,000 IU oral vitamin D3 (cholecalciferol) for 21 days resulted in a statistically significant increase in cathelicidin expression in the AD lesions [45]. It was not determined if supplementation improved immunologic outcomes in these studies; therefore, future randomized controlled trials are needed to establish an immune enhancing role for vitamin D supplementation and to determine optimal circulating levels.

## Competing interests

BD, TM and JC are employed by USANA Health Sciences.

## Authors’ contributions

BD, JC, BF and AFG conceived of and designed the study. BD, TM, JC, BF, NB and AFG supervised and/or were directly involved in the acquisition of data. TB, BD and AFG analyzed the data. TB, BD and AFG drafted the initial manuscript and all co-authors reviewed the manuscript for important intellectual content and read and approved the final version.
